# Correlation Between Pediatrician Supply and Public Health in Japan as Evidenced by Vaccination Coverage in 2010: Secondary Data Analysis

**DOI:** 10.2188/jea.JE20140121

**Published:** 2015-05-05

**Authors:** Rie Sakai, Günther Fink, Wei Wang, Ichiro Kawachi

**Affiliations:** 1 Department of Social and Behavioral Sciences, Harvard School of Public Health, Boston, MA, USA; 2 Department of Medical Education, Juntendo University School of Medicine, Tokyo, Japan; 3 Department of Pediatrics and Adolescent Medicine, Juntendo University School of Medicine, Tokyo, Japan; 4 Department of Global Health and Population, Harvard School of Public Health, Boston, MA, USA; 5 Department of Sleep Medicine, Brigham and Women’s Hospital, Boston, MA, USA

**Keywords:** human resources, physician supply, vaccination coverage, healthcare utilization, Japan

## Abstract

**Background:**

In industrialized countries, assessment of the causal effect of physician supply on population health has yielded mixed results. Since the scope of child vaccination is an indicator of preventive health service utilization, this study investigates the correlation between vaccination coverage and pediatrician supply as a reflection of overall pediatric health during a time of increasing pediatrician numbers in Japan.

**Methods:**

Cross-sectional data were collected from publicly available sources for 2010. Dependent variables were vaccination coverage for measles and diphtheria, pertussis, and tetanus (DPT) by region. The primary predictor of interest was number of pediatricians per 10 000-child population (pediatrician density) at the municipality level. Multivariate logistic regression models were used to estimate associations of interest, conditional on a large range of demographic and infrastructure-related factors as covariates, including non-pediatric physician density, total population, per capita income, occupation, unemployment rate, prevalence of single motherhood, number of hospital beds per capita, length of roads, crime rate, accident rate, and metropolitan area code as urban/rural status. The percentage of the population who completed college-level education or higher in 2010 was included in the model as a proxy for education level.

**Results:**

Pediatrician density was positively and significantly associated with vaccination coverage for both vaccine series. On average, each unit of pediatrician density increased odds by 1.012 for measles (95% confidence interval, 1.010–1.015) and 1.019 for DPT (95% confidence interval, 1.016–1.022).

**Conclusions:**

Policies increasing pediatrician supply contribute to improved preventive healthcare services utilization, such as immunizations, and presumably improved child health status in Japan.

## INTRODUCTION

Maintaining an optimal supply and distribution of physicians^1^^–^^15^ is a critical objective for countries that strive for equitable healthcare services for their citizens. In Japan, attempts have been made to raise medical student quotas to increase the number of physicians. These policy interventions are based on the assumption that increasing the number of physicians will alleviate physician shortages, thus improving population health.^16^ However, evidence regarding the causal effect of physician numbers on population health has shown mixed results in industrialized countries,^17^^–^^23^ and substantiation of benefits from increased numbers is particularly lacking in Japan. Additionally, most previous investigations have been limited by their focus on the total supply of physicians, without regard to specialty. Two notable exceptions are Cochrane et al,^18^ who used the disaggregated number of pediatricians and obstetricians in addition to the total number of physicians, and Goodman et al,^23^ who focused on the supply of neonatologists to test the relationship with neonatal mortality.

Although previous cross-national studies^24^^–^^26^ have already demonstrated a relationship between healthcare workers and vaccination coverage, we believe that our within-country study contributes to the existing literature for the following reasons. First, as Speybroeck et al^27^ noted, the qualifications, training, classification, and roles of healthcare workers vary widely from country to country. For example, in Japan, only physicians can administer vaccinations. Therefore, as argued by Mitchell et al,^25^ a within-country analysis avoids such limitations and can provide somewhat stronger evidence regarding the association between the supply of healthcare workers and the provision of preventive healthcare services. Second, previous studies mainly focused on developing countries.^24^^–^^26^ Clearly, physician supply studies from developing countries cannot be generalized to industrialized countries, primarily because the health-related issues in industrialized countries are different from those in developing countries.^28^ For example, infectious diseases are still major causes of death in developing countries, whereas most causes of death in industrialized countries are attributed to chronic diseases.^28^ In addition, the extent of physician shortages in developing countries is substantially different from that in industrialized countries. Eckhert^29^ estimated that the average physician-per-100 000-population ratio in sub-Saharan Africa was 10, compared to 240 in the United States.

A within-country study in an industrialized nation conducted by LeBaron et al^30^ showed that pediatrician density is positively associated with vaccination coverage; however, the effective sample size of this study was 51 because the 50 U.S. states plus Washington D.C. were used as the unit of analysis, which limited the predictors in the model. Furthermore, as the authors noted, the interpretation of state-based findings was complicated because of the diversity of healthcare environments within each study locality (eg, rural southern Illinois versus inner-city Chicago).

The objective of this study is to investigate the association between the pediatrician supply and preventive health services utilization in Japan in 2010, employed here as an indicator of pediatric population health. The study focuses on vaccination coverage as an outcome measure because vaccination coverage is considered one of the most cost-effective preventive health measures for children^31^^,^^32^ and is most directly linked to pediatricians. Our study has a fairly large sample size of about 1700 municipalities.

## MATERIALS AND METHODS

### Study background

Japan had 47 prefectures with 1742 municipalities at the time of this study. Municipalities are the basic geographical units of administration and were used as the unit of analysis in this study.

Unlike many other countries, the Preventive Vaccination Law in Japan offers a system that is entirely voluntary rather than mandatory.^33^ The legislation classifies vaccinations into two groups: “scheduled vaccines” and “elective vaccines”. Scheduled vaccines are administered free of charge by the local health sector, and target ages are clearly defined by the law, whereas the elective vaccines require individuals to pay out-of-pocket.^33^ As of 2010, scheduled vaccines include poliomyelitis (polio), tuberculosis, diphtheria, pertussis, tetanus, measles, rubella, Japanese encephalitis, and influenza. Pursuant to the Community Health Act, vaccination coverage is officially recorded for scheduled vaccinations in public health registries.^33^

Typically, vaccinations other than polio are administered by physicians in hospitals or clinics,^34^ while polio vaccinations are administered twice annually (spring and fall) at public healthcare centers. In 2010, the National Childhood Immunization Program in Japan recommended that the diphtheria, pertussis, and tetanus (DPT) vaccine be administered three times by the age of 1, followed by a booster between 12 and 18 months after the third dose of DPT, and the measles-rubella (MR) vaccination be administered a total of four times: once at the age of 1, once before entering primary school, once in the seventh grade, and once during high school.^35^ MR vaccinations for seventh graders and high-school students were only administered between 2008 and 2013. In this study, the third dose of the DPT vaccine and the MR vaccination coverage for 1-year-olds were used as an outcome variable.

### Data

The data used in this study were compiled from multiple sources. Data for the number of physicians within each type of municipality were obtained from the Survey of Physicians, Dentists, and Pharmacologists (Physician Survey),^36^ which is conducted every 2 years by the Ministry of Health, Labour and Welfare (MHLW). All licensed physicians are expected to complete this survey and register their working addresses and specialties under the Medical Practitioners Law.^37^ The estimated registration rate is reported to be between 87% and 90%.^38^ The data used in this paper stem from the 2010 survey round. The year of 2010 was selected as the study year because broad ranges of variables were available from the Japanese national census, which is conducted every 5 years. Data on local populations by age group were obtained from the resident registers compiled by the Ministry of Internal Affairs and Communications (MIAC) in March of each year, to reflect data from April 2010 through March 2011.^39^ Data for MR vaccination coverage were obtained from public health registries containing the implementation status of the four MR vaccinations.^40^ Data for DPT vaccinations were derived from the Report on Regional Public Health Services and Health Promotion Services.^41^

Demographic data for the following seven variables were obtained from the 2010 Japanese Census: (1) total population, (2) income, (3) the percentage of the population who completed college-level education or higher among all graduates in 2010 (as a proxy for educational level), (4) the percentage of the population who were white-collar workers, (5) unemployment rate, (6) the prevalence of single motherhood, and (7) metropolitan area code.^42^^–^^44^ Data for 2010 chronicling the length of roads, as well as numbers of beds, crimes, and accidents, were obtained from annual reports of social welfare indicators from the MIAC’s Statistics Bureau.^45^

### Empirical approach

The primary outcome variables of interest were vaccination coverage for MR and DPT, each defined as the number of children who received the vaccination divided by the number of children who were expected to be vaccinated. For MR, both numbers are reported in the registry.^40^ For DPT, the official announcement of vaccination coverage from the MHLW uses the number of children who received the vaccination from the *Report of Community Health and Health of the Elderly* and calculates the estimated number of children who were expected to be vaccinated from the following equation (MHLW, personal communication)^46^:

$$\begin{array}{l} \text{(Estimated number of children expected to be vaccinated)}\\ \;=\text{(population at age 0)}\times 9/12\\ \;\;+\text{(population at age 1)}\times 3/12 \end{array}$$

This study follows the above method. The premise behind this calculation is that the target population for DPT is older than 3 months old, with completion expected within 1 year.

Some municipalities showed vaccination coverages higher than 100%. One possible explanation of this anomaly is that some children expecting to be vaccinated in the previous year were vaccinated during the study year. In this case, the vaccination coverage was truncated to 100%; as a robustness check, municipalities with more than 100% vaccination coverage were excluded from the analyses. Municipalities with no children scheduled to be vaccinated were also excluded from the analyses.

Pediatrician density, which is defined as number of pediatricians per 10 000 children, was used as the primary predictor of interest. Children are defined as the population under 15 years of age because this age group is exclusively treated by pediatricians in Japan. Other independent variables were chosen based on existing evidence showing associations with vaccination coverage^26^^,^^30^^,^^47^^–^^58^ or physician supply.^59^^–^^62^ The variables introduced into the final model were obtained using backward elimination.^63^^–^^65^ Land area and pediatrician density remained in the model throughout the model building process because pediatricians are more thinly-spread and the distance between pediatricians and children is greater in municipalities with larger geographical areas.^24^^–^^26^ We developed separate models for the two different vaccines to allow for the possibility that predictors that affect the relationship between pediatrician density and the two vaccines might differ, as Anand et al^24^ has noted.

Table 1 shows each of the candidate variables in the models. In addition to the variables described in Table 1, the density of other types of physicians was taken into account, since all physicians can administer vaccines to children. The definition of primary care in Japan is ambiguous, and there is no standard specialty term or professional organization that corresponds to the internist, family physician, or the general practitioner in the United States or United Kingdom.^66^ As such, pediatricians play a major role in providing pediatric care, so general practitioner or family physician density was not included in the model.

**Table 1.** Variables selected as candidates in the regression models

Variables		Definition	
Total population		Number of registered residents	
Per capita income		Income per number of registered residents	
Percentage of the population with a college-level education		As a proxy for education level	
Percentage of the working population who are white-collar workers		As a proxy for occupation	
		•	number of professionals, technical workers and managers and administrators per number of all individuals currently in the labor force (workforce)
Unemployment rate		Number of job-seekers per workers and job-seekers	
Prevalence of single motherhood		Number of households with single mothers per number of households with children	
Number of hospital beds per total population		As a proxy for health infrastructure	
Total length of roads		As a proxy for accessibility of vaccination site	
Crime rate		Number of crimes per number of registered residents	
Accident rate		Number of accidents per number of registered residents	
Urban/rural status		Metropolitan area code defined by the Ministry of Internal Affairs and Communications	
1)	Central cities in metropolitan areas		
2)	Surrounding municipalities of central cities in metropolitan areas		
3)	Other municipalities (rural areas)		

Furthermore, we created a dichotomous indicator designated *PED_dummy*. *PED_dummy* equals 1 if municipalities had at least one pediatrician and equals 0 otherwise. There were some municipalities that did not have any pediatricians at the time of the study. Children at the municipalities without pediatricians typically would visit other physicians, and therefore, the utilization of other types of physicians was assumed to be different between municipalities with and without pediatricians. As a result, *PED_dummy* and an interaction term between *PED_dummy* and the density of other types of physicians were included in the model.

To describe urban/rural status, we employed the metropolitan area code defined by the MIAC, which classifies municipalities into the following five categories: (1) central cities of major metropolitan areas, (2) central cities of metropolitan areas, (3) surrounding municipalities of central cities of major metropolitan areas, (4) surrounding municipalities of central cities of metropolitan areas, and (5) other municipalities. In this study, major metropolitan and metropolitan areas were combined into one category because there were only six central cities of metropolitan areas among 1742 municipalities, leaving three categories: (1) central cities in metropolitan areas, (2) surrounding municipalities of central cities in metropolitan areas, and (3) other municipalities. To avoid multicollinearity, a composite index of socioeconomic indicators (SES composite index) was created from socioeconomic variables for education, occupation, and income. The index was based on a factor analysis of the percentage of the population who completed college-level education or higher among all graduates in 2010, the percentage of the population who were white-collar workers, the unemployment rate, and per capita income. Factor scores, formulated by a principal component analysis with varimax rotation, were used to construct a composite index to represent each aspect of socioeconomic status for the study units. Regional dummy variables for prefectures were also introduced into the model to control for prefecture effects.

Two additional sets of sensitivity analyses were performed for the models not containing municipalities with more than 100% vaccination coverage. First, the population under the age of 5 was used to calculate pediatrician density because this age group tends to have greater demand for pediatric medical services.^59^ Second, we used the fixed-effects model below to examine the effect of pediatrician supply on vaccination coverage. We employed municipality and year fixed effects to control for time-invariant and municipality-specific differences as follows:

$$\begin{array}{l} Vaccine_{at}=\beta_{0}+\beta_{1}PED_{at}+\chi_{at}\gamma \\ \;\;\;\;\;+\sum_{a=2}^{1738}\lambda_{a}I_{a}+\sum_{t=2004}^{2010}\alpha_{t}I_{t}+\varepsilon_{at} \end{array}$$

where *Vaccine* refers to vaccination coverage in municipality *a* at year *t*; *PED* refers to pediatrician density in municipality *a* at year *t*; X is a vector of time-varying characteristics at municipality level; α*_t_* is year fixed effects, which are year-specific effects common to all municipalities (captured by year dummies); λ_a_ is municipality fixed effects, which absorb all municipality-specific time-invariant effects; and ε is a municipality and year-specific random-error term. α*_t_* captures general secular and countrywide trends. In addition to the municipality and year fixed effects, the following four variables were introduced into the model as time-varying characteristics: (1) total population, (2) density of other types of physicians, and (3) per capita income by year and municipality. *PED_dummy* and an interaction term between *PED_dummy* and the density of other types of physicians were also included in the model.

Data for DPT vaccinations between 2001 and 2012 were available, while data for MR vaccinations between 2010 and 2012 were available. Combined with the Surveys of Physicians, Dentists, and Pharmacologists, which are conducted every 2 years, we were able to perform this sensitivity analysis only for DPT vaccination. Additionally, DPT was in the transitional phase to DPT-IPV in 2012. Therefore, the following five time points were used for the analysis: 2002, 2004, 2006, 2008, and 2010. Furthermore, population by age at the municipality level was only available for the year 2010; therefore, we estimated the number of children who were expected to be vaccinated using the following equation:

$$\begin{array}{l} \text{(Estimated number of children expected}\\ \text{to}\;\text{be}\;\text{vaccinated}\;\text{at}\;{\text{year}}_{t})\\ \;=(\text{the}\;\text{number}\;\text{of}\;\text{births}\;\text{at}\;{\text{year}}_{t})\times \text{9/12}\\ \;\;+(\text{the}\;\text{number}\;\text{of}\;\text{births}\;\text{at}\;{\text{year}}_{\left(\text{t-1}\right)})\times \text{3/12} \end{array}$$

The premise behind this calculation is that the number of births at year t represents population at age 0 at year t and the number of births at year (t − 1) represents population at age 1. From 2002 to 2010, Japan underwent administrative reorganization through a large-scale merging of municipalities, resulting in the decrease of the total number of municipalities during the study period. Every data set was adjusted for the new municipal boundaries by merging former smaller municipalities into later larger ones.

### Statistical analysis

Descriptive statistics of all variables were presented as means with standard deviations (SDs). Logistic regression models were used to investigate the association between pediatrician density and vaccination coverage at the municipal level.

A two-tailed *P* of less than 0.05 was considered statistically significant. All analyses were performed using *SAS* 9.2 (SAS Institute, Inc., Cary, NC, USA).

## RESULTS

There were 1742 municipalities in 2010. A total of 7 municipalities had missing data for measles, and 46 municipalities had missing data for DPT, due mainly to the Tohoku earthquake in March 2011. Combined with those municipalities with no children expected to be vaccinated at the time of this study, 1733 municipalities were used for the analysis of measles vaccination, and 1691 municipalities were used for the analysis of DPT. Table 2 presents means and SDs for all dependent and independent variables. Table 3-1 shows the aggregate level change in dependent and independent variables, and Table 3-2 shows means and SDs for all dependent and independent variables from 2002 and 2010 that were used in the fixed-effects model.

**Table 2.** Descriptive statistics using municipality as a unit of analysis

	Mean	SD
Measles vaccination coverage	94.56	(11.80)
DPT vaccination coverage	96.32	(25.05)
Pediatrician density^a^	5.24	(7.03)
Population (thousand)	73.11	(180.93)
Per capita income (thousand US$)^b^	10.41	(3.29)
Percentage of the population with a college-level education	0.12	(0.06)
Percentage of the population who are white-collar workers^c^	13.52	(3.11)
Unemployment rate^d^	6.3	(2.17)
The incidence of single motherhood^e^	0.06	(0.03)
Number of hospital beds/1000 population	10.96	(10.51)
Length of roads (km)	0.69	(0.8)
Crime rate	8.85	(5.84)
Accident rate	4.64	(2.61)
Land area (km^2^)	214.37	(247.65)
Other physician density^f^	13.96	(15.22)

SD, standard deviation.

^a^The number of pediatricians per 10 000 children under the age of 15.

^b^Japanese yen was converted into US$ using the rate that applied in April 2014 of approximately 105 Japanese yen per US$.

^c^Percentage of population who are white-collar workers defined as the number of professionals, technical workers and managers and administrators per the total number of individuals currently in the labor force (workforce).

^d^The number of unemployed individuals per workforce.

^e^The number of household with single motherhood per the number of household with children.

^f^The number of other types of physicians per 10 000 population.

**Table 3-1.** The aggregate level change in dependent and independent variables from 2002 and 2010

	2002	2004	2006	2008	2010
DPT vaccination coverage	97.68	98.32	99.64	103.34	101.77
Pediatrician density^a^	7.99	8.25	8.38	8.81	9.31
Other physician density^b^	18.59	19.08	19.59	20.20	20.82
Total population (million)	126.50	126.80	127.10	127.10	127.10
Per capita income (thousand US$)^c^	13.83	13.19	14.00	14.34	13.18

^a^The number of pediatricians per 10 000 population under the age of 15.

^b^The number of physicians other than pediatricians per 10 000 population.

^c^Japanese yen was converted into US$ using the rate that applied in April 2014 of approximately 105 Japanese yen per US$.

**Table 3-2.** Means and standard deviations of dependent and independent variables from 2002 and 2010

		2002	2004	2006	2008	2010
DPT vaccination coverage	mean	92.77	92.67	95.42	96.61	96.44
	(SD)	(12.5)	(11.54)	(9.32)	(9.26)	(8.85)
Pediatrician density^a^	mean	4.83	5.10	5.17	5.43	5.54
	(SD)	(6.64)	(6.90)	(7.12)	(7.76)	(7.53)
Other physician density^b^	mean	12.97	13.20	13.39	13.62	13.87
	(SD)	(14.39)	(14.57)	(14.9)	(15.12)	(15.71)
Total population (thousand)	mean	72.76	72.97	73.10	73.11	73.11
	(SD)	(174.69)	(176.46)	(178.07)	(179.55)	(180.93)
Per capita income (thousand US$)^c^	mean	11.39	10.73	11.19	11.29	10.41
	(SD)	(3.75)	(3.13)	(3.51)	(3.81)	(3.29)

SD, standard deviation.

^a^The number of pediatricians per 10 000 population under the age of 15.

^b^The number of physicians other than pediatricians per 10 000 population.

^c^Japanese yen was converted into US$ using the rate that applied in April 2014 of approximately 105 Japanese yen per US$.

Table 4 presents the results from the regression analyses. Based on the model building procedure, all the candidate independent variables were included in the final model for the analyses of DPT vaccinations; moreover, the ones without length of road and the prevalence of single motherhood were included in the final model for the analyses of MR vaccinations. Pediatrician density was positively associated with vaccination coverage for both MR and DPT in both the univariate and multivariate analyses (*P* < 0.001 for both vaccinations). The fully-adjusted models displayed in Table 4 suggest that, on average, a unit increase in pediatrician coverage was associated with increased odds of 1.012 for MR (95% CI, 1.010–1.015) and 1.019 for DPT (95% CI, 1.016–1.022).

**Table 4.** Estimated odds ratios for the increase in vaccination coverage associated with an increase of one pediatrician per 10 000 children in Japan

	OR [95% CI]	*P* value	Adjusted^a^ OR [95% CI]	*P* value
Measles-Rubella (*n* = 1733)				
	1.022 [1.021–1.024]	<0.001	1.012 [1.010–1.015]	<0.001
DPT (*n* = 1691)				
	1.013 [1.011–1.014]	<0.001	1.019 [1.016–1.022]	<0.001

CI, confidence interval; DPT, diphtheria, pertussis, and tetanus; OR, odds ratio.

^a^The models included the following control variables: total population, a composite index of socioeconomic indicators created from socioeconomic variables for the percent of the population with a college-level education, the percent of white-collar workers, the unemployment rate, and per capita income, the incidence of single motherhood, number of hospital beds per 1000 population, length of roads, crime rate, accident rate, land area, *PED_dummy* which equals 1 if municipalities had at least one pediatrician and equals 0 otherwise, and an interaction term between *PED_dummy* and the density of other types of physicians and regional dummy variables for prefectures for DPT and the ones without length of road and the prevalence of single motherhood for Measles-Rubella.

Table 5 shows the results from the robustness check that excludes municipalities with more than 100% vaccination coverage. Table 6 shows the results from the robustness check that uses population under the age of 5 years to calculate pediatrician density. Table 7 shows the results from the robustness check that uses population under the age of 5 years to calculate pediatrician density and excludes municipalities with more than 100% vaccination coverage. All robustness checks concur that pediatrician density was positively associated with vaccination coverage for both vaccines in both the univariate as well as multivariate analyses (*P* < 0.001 for all analyses). Table 8 shows the results from the regression model with municipality and year fixed effects. When considering the national average association, a unit increase in pediatrician coverage was associated with increased odds of 1.025 for DPT (95% CI, 1.021–1.029), after adjustment for all other variables.

**Table 5.** Estimated odds ratio for the increase in vaccination coverage associated with an increase of 1 pediatrician per 10 000 children in Japan, excluding municipalities with more than 100% vaccination coverage

	OR [95% CI]	*P* value	Adjusted^a^ OR [95% CI]	*P* value
Measles-Rubella (*n* = 1450)				
	1.021 [1.019–1.023]	<0.001	1.008 [1.006–1.011]	<0.001
DPT (*n* = 1263)				
	1.016 [1.014–1.018]	<0.001	1.011 [1.008–1.014]	<0.001

CI, confidence interval; DPT, diphtheria, pertussis, and tetanus; OR, odds ratio.

^a^The models included the following control variables: total population, a composite index of socioeconomic indicators created from socioeconomic variables for the percent of the population with a college-level education, the percent of white-collar workers, the unemployment rate, and per capita income, the incidence of single motherhood, number of hospital beds per 1000 population, length of roads, crime rate, accident rate, land area, *PED_dummy* which equals 1 if municipalities had at least one pediatrician and equals 0 otherwise, and an interaction term between *PED_dummy* and the density of other types of physicians and regional dummy variables for prefectures for DPT and the ones without length of road and the prevalence of single motherhood for Measles-Rubella.

**Table 6.** Estimated odds ratio for the increase in vaccination coverage associated with an increase of 1 pediatrician per 10 000 children under the age of 5 in Japan

	OR [95% CI]	*P* value	Adjusted^a^ OR [95% CI]	*P* value
Measles-Rubella (*n* = 1733)				
	1.009 [1.008–1.09]	<0.001	1.006 [1.005–1.007]	<0.001
DPT (*n* = 1691)				
	1.005 [1.005–1.006]	<0.001	1.008 [1.007–1.009]	<0.001

CI, confidence interval; DPT, diphtheria, pertussis, and tetanus; OR, odds ratio.

^a^The models included the following control variables: total population, a composite index of socioeconomic indicators created from socioeconomic variables for the percent of the population with a college-level education, the percent of white-collar workers, the unemployment rate, and per capita income, the incidence of single motherhood, number of hospital beds per 1000 population, length of roads, crime rate, accident rate, land area, *PED_dummy* which equals 1 if municipalities had at least one pediatrician and equals 0 otherwise, and an interaction term between *PED_dummy* and the density of other types of physicians and regional dummy variables for prefectures for DPT and the ones without length of road and the prevalence of single motherhood for Measles-Rubella.

**Table 7.** Estimated odds ratio for the increase in vaccination coverage associated with an increase of 1 pediatrician per 10 000 children under the age of 5 in Japan, excluding municipalities with more than 100% vaccination coverage

	OR [95% CI]	*P* value	Adjusted^a^ OR [95% CI]	*P* value
Measles-Rubella (*n* = 1450)				
	1.008 [1.008–1.009]	<0.001	1.004 [1.003–1.005]	<0.001
DPT (*n* = 1263)				
	1.006 [1.006–1.007]	<0.001	1.005 [1.004–1.006]	<0.001

CI, confidence interval; DPT, diphtheria, pertussis, and tetanus; OR, odds ratio.

^a^The models included the following control variables: total population, a composite index of socioeconomic indicators created from socioeconomic variables for the percent of the population with a college-level education, the percent of white-collar workers, the unemployment rate, and per capita income, the incidence of single motherhood, number of hospital beds per 1000 population, length of roads, crime rate, accident rate, land area, *PED_dummy* which equals 1 if municipalities had at least one pediatrician and equals 0 otherwise, and an interaction term between *PED_dummy* and the density of other types of physicians and regional dummy variables for prefectures for DPT and the ones without length of road and the prevalence of single motherhood for Measles-Rubella.

**Table 8.** Results from multivariate regression model with municipality and year fixed effects

	Estimate	SE	95% CI		*P*
**Pediatrician density**^a^	**1.025**	**0.002**	**1.021**	**1.029**	**<0.001**
Other physician density^b^	1.017	0.004	1.008	1.025	<0.001
*PED_dummy*^c^	0.976	0.056	0.873	1.090	0.66
Interaction term between other physician density and *PED_dummy*	1.006	0.004	0.997	1.014	0.18
Total population (hundred thousand)	1.005	0.000	1.005	1.006	<0.001
Per capita income (thousand US$)^d^	0.928	0.004	0.921	0.936	<0.001

CI, confidence interval; SE, standard error.

^a^The number of pediatricians per 10 000 population under the age of 15.

^b^The number of other physicians per 10 000 population.

^c^A function that equals 1 if municipalities had at least one pediatrician and equals 0 otherwise.

^d^Japanese yen was converted into US$ using the rate that applied in April 2013 of approximately 105 Japanese yen per US$.

## DISCUSSION

The present analysis suggests a positive association between pediatrician density and vaccination coverage in Japan. Like other industrialized countries, Japan has experienced a large reduction in the mortality rates caused by infectious diseases.^67^^–^^76^ Although mortality due to infectious diseases is low, there are still cases of infectious diseases in Japan.^77^^,^^78^ Specifically, in the past 10 years, approximately 100 cases of tetanus and 20 000 estimated cases of pertussis (an estimate because Japan only has sentinel surveillance systems for pertussis) were reported.

For measles, Japan used sentinel surveillance systems between 1999 and 2007, which were replaced with a nationwide case-based reporting system in 2008. After 2008, approximately 500 cases of measles were reported each year. However, it is important to note that a measles outbreak (predominantly in young adults) occurred in Japan during 2007 and 2008, with 11 015 reported cases of measles. Measles is one of the leading causes of death among young children in the world, even though a safe and cost-effective vaccine is available.^79^

Evidence shows that Japan still experiences cases of preventable infectious diseases. An expert advisory panel convened by the World Health Organization (WHO) to assess the feasibility of measles eradication concluded that measles can and should be eradicated.^80^ The Japanese government set a goal to eradicate measles by 2012 and to be certified by WHO as a country with no measles by 2015.^81^ To achieve this goal, the government determined that vaccination coverage must exceed 95%,^81^^,^^82^ with this goal achieved in 2010, 2011, and 2012 (most recent data at this point).^46^ However, in 2014, more than 300 cases were reported during the first half of the year, which is the highest number of cases over the same months of the year since the outbreak in 2008. Among the cases in 2014, almost half were not vaccinated. It is suggested that the measles virus was brought from other countries by travelers.^83^ Due to globalization, importing measles virus from other countries is always possible. Although vaccination coverage for measles at the national level exceeds 95%,^46^ almost half of the municipalities in Japan have not reached 95% coverage. Given the cases in 2014, even though the vaccination coverage at the national level exceeds 95%, it is important to increase vaccination coverage at the municipality level. With preventable infectious diseases still present, increasing the number of pediatricians and improving vaccination coverage may continue to improve the health status of children in Japan.

We would like to note that increasing the total number of pediatricians does not ensure that the supply of pediatricians in areas of low vaccination coverage will be enhanced. Sakai et al^84^ showed that the number of pediatricians increased in areas where the pediatrician density was already high after 2004, when Japan launched the national matching system, rather than in the areas where more pediatricians would be needed. To improve the health status of children, balancing the spatial distribution of physicians based on the regional needs should be taken into account, along with overall increases in the number of physicians.

There are some limitations to this study. First, there are concerns regarding the quality of the public data on the number of children actually receiving vaccinations and the validity of the denominator to calculate vaccination coverage each year. A total of 283 (16.3%) and 428 (25.3%) municipalities had more than 100% coverage rates for MR and DPT, respectively. This suggests that many parents do not finish vaccination of their child for MR (one dose) and DPT (three doses) by the age of 1 year. Guidelines recommend that the first dose of DPT should be administered after 3 months old, and that there should be a 3–8 week interval between the next two doses. Additionally, a booster inoculation is recommended between 12 and 18 months after the completion of the third dose of DPT. Since local governments cover the cost until the child reaches 90 months (7.5 years) of age, it is not surprising that some parents do not finish the third dose of DPT by age 1. However, families not finishing the third dose of DPT or the first dose of MR by age 1 is a common occurrence that happens every year and is not a special event in this study period. Therefore, although the denominators in the equation may not correlate precisely to the vaccination coverage, we believe these discrepancies are not great enough to alter the indications of beneficial effects of pediatrician supply on vaccination coverage. In addition, most of these municipalities were rather small, which makes estimation errors less likely. To make sure our results were not affected by outliers, we also conducted the analysis without these municipalities, which did not alter the overall results.

Second, the cross-sectional nature of the data limits our ability to make causal inference. While we include an extensive set of municipality covariates, some residual confounding bias cannot be excluded. For instance, it is possible that municipalities with higher vaccination demand (or stronger preferences for vaccinations) make more efforts to recruit pediatricians. Given that pediatricians can freely choose the location of their practice, this scenario is possible, but does not seem very likely. Furthermore, for DPT, we used panel data and showed the robustness of the results.

Third, we have used length of roads as a proxy for accessibility in this study. Although public transportation is another major factor that will affect accessibility, Japan has better-organized public transportation systems than many other countries. However, data for public transportation were not available and could not be included in the model.

Fourth, we were not able to analyze the cost-effectiveness of increased number of physicians on vaccination coverage. Especially given the small increase in vaccination coverage that a one-unit increase in pediatrician density can make, there are less expensive ways to increase vaccination coverage; therefore, the cost of the extra pediatricians might not be worth the increase in vaccination coverage. The Guide to Community Preventive Services^85^ says that the available studies do not provide sufficient evidence to determine if healthcare-system-based interventions, including expanded access in healthcare, are effective or not. We feel that evaluation of the economic efficiency of increasing pediatrician supply is beyond the scope of this paper.

Despite these limitations, we believe that our study contributes to the debate surrounding the impact of pediatrician supply on population health for the following reasons. First, children and their parents must go to medical facilities (hospitals, clinics, or public health centers) for vaccinations within the municipality where they reside. In Japanese culture, many women go back to their hometown (where their mother lives) to give birth, and most local governments cover the cost for vaccination in other municipalities for this case. The percent of these women varies between 8% and 37%, depending on reports and the culture of the region where they reside.^86^^,^^87^ The length of stay also varies. Ohga et al^86^ studied 506 couples and reported that the length of stay (mean ± SD) in the mothers’ hometowns was 32.9 ± 19.0 days among those who went back to their hometown for childbirth. Applied to the result of Ohga et al’s study, it is likely that most mothers and babies have already returned to their own municipalities by the time the babies have received the first DPT and MR inoculations, as the recommended age for the first administration of the DPT vaccine is 3 months old and the recommended age for the first administration of the MR vaccine is 12 months old. Therefore, the provision of healthcare services across municipalities’ borders is not an issue in this study. Second, compared to other studies, this study considered a broader range of independent variables besides independent variables for healthcare workers, including all the socioeconomic indicator variables in Table 1.^47^^,^^48^^,^^50^^,^^51^^,^^53^ In addition, to control for a number of municipal characteristics, we employed a regression model with municipality and year fixed effects, which control underlying unmeasured municipal characteristics, and this sensitivity analysis also showed the beneficial effect of pediatrician supply on DPT vaccination coverage. Third, previous studies^24^^–^^26^^,^^30^ used the aggregated total number of physicians as an independent variable. By analyzing the physicians that are most likely to provide pediatric healthcare, this current study provides a more accurate picture of the impact of pediatrician supply on child health status.

### Conclusion

The results of this study suggest that pediatrician coverage is positively associated with vaccination rates of children in Japan. Given the relationship between child health and the utilization of important preventive health services like vaccinations, continued government efforts to sustain high levels of pediatrician supply across Japanese municipalities appear desirable from a public and child health perspective.
